# A Pilot Study Evaluating the Impact of an Algorithm-Driven Protocol on Guideline-Concordant Antibiotic Prescribing in a Rural Primary Care Setting

**DOI:** 10.3390/pharmacy13010030

**Published:** 2025-02-19

**Authors:** Arinze Nkemdirim Okere, Anthony Ryan Pinto, Sandra Suther, Patrick Ten Eyck

**Affiliations:** 1 College of Pharmacy, The University of Iowa, 180 South Grand Ave, 366B College of Pharmacy Building, Iowa City, IA 52242, USA; 2 Community Health Northwest Florida Community-Based Pharmacy Residency Program, Florida A&M University, 2315 W Jackson St, Pensacola, FL 32505, USA; anthony.pinto@famu.edu; 3 College of Pharmacy and Pharmaceutical Sciences, Institute of Public Health, Florida A&M University, 1415 Martin Luther King Jr. BLVD, Tallahassee, FL 32307, USA; sandra.suther@famu.edu; 4 Institute for Clinical and Translational Science, Department of Biostatistics, University of Iowa, 200 Hawkins Drive, SW44-M GH, Iowa City, IA 52246, USA; patrick-teneyck@uiowa.edu

**Keywords:** antibiotic stewardship program, rural health, underserved population, antibiotics, pharmacy

## Abstract

Approximately 2.8 million cases of bacterial antimicrobial resistance (AMR) infections result in over 35,000 deaths annually in the U.S. AMR is driven largely by inappropriate prescribing of antibiotics, especially in clinics serving rural communities or underserved populations. Antibiotic Stewardship Programs (ASPs) improve prescribing practices, but many rural clinics lack fully functional ASPs. This pilot study evaluated the impact of an algorithm-driven protocol on antibiotic prescribing in a rural primary care setting. We conducted a pre–post quasi-experimental study at a Federally Qualified Health Center (FQHC), focusing on upper respiratory infections, urinary tract infections, and sexually transmitted infections. Eligible patients were enrolled in the study during their primary care visits. The primary outcome was the frequency of guideline-concordant treatment, analyzed using descriptive statistics and Chi-square tests. Among 201 patients (101 pre-intervention, 100 post-intervention), the pre-intervention group consisted of 77% females and 47% African Americans, while the post-intervention group consisted of 72% females and 46% African Americans. The intervention was associated with a 12.6% decrease in the number of antibiotic prescriptions discordant with clinical guidelines (37.6% to 25%) from the pre- to post-intervention periods. This corresponded to an odds ratio of 0.55 (95% CI: 0.30–1.01, *p* = 0.054). Although not statistically significant at α = 0.05, this numerical decrease suggests potential benefits of algorithm-driven protocols in improving antibiotic stewardship in resource-limited settings. Longer study periods may further elucidate these benefits.

## 1. Introduction

Each year in the United States, approximately 2.8 million cases of antimicrobial-resistant bacterial infections result in over 35,000 deaths [1]. A significant driver of antimicrobial resistance (AMR) is the inappropriate prescribing of antibiotics, particularly in outpatient settings, where over 50% of prescriptions are deemed unnecessary or inappropriate [2,3,4]. This issue is especially pronounced in clinics serving rural or underserved populations [5,6].

Fortunately, antibiotic stewardship programs (ASPs) have been shown to significantly improve antibiotic prescribing [7,8,9], yet they remain largely absent in clinics serving rural communities or underserved populations. Despite the Centers for Disease Control and Prevention (CDC) recognizing inappropriate antibiotic prescribing as a significant issue in these communities—contributing to antimicrobial resistance (AMR) and *Clostridioides difficile* infections—the full extent of the problem remains inadequately understood [10]. A 2020 survey of Vizient member hospitals found that while 88% of inpatient institutions had functional ASPs, only 7% of ambulatory practices implemented them, most of which were in urban areas [11]. Given the limited healthcare access and resources in rural settings, the prevalence of ASPs in these communities is likely even lower. This gap highlights the urgent need for targeted strategies to curb antibiotic misuse and overuse in rural and underserved populations.

Several randomized controlled trials have demonstrated that strategies such as point-of-care testing, communication skills training, clinical decision support, and individualized audit-and-feedback for providers can effectively reduce unnecessary antibiotic use in primary care clinics [12,13,14,15,16]. However, these strategies often require substantial technical resources that may not be readily available or sustainable in resource-limited settings, such as clinics serving rural communities or underserved populations. Understanding these challenges is crucial to developing feasible and effective antimicrobial stewardship programs (ASPs) tailored to these environments.

To gain insight into the perspectives of both patients and clinicians regarding the implementation of ASPs in such settings, we conducted a survey study in a primary care center serving rural communities and underserved populations. The findings revealed that both patients and healthcare providers expressed openness to the integration of ASPs, highlighting a willingness to adopt stewardship initiatives despite existing resource constraints [17,18]. Furthermore, as part of this endeavor to improve antibiotic prescribing in resource-limited clinics, we evaluated the impact of integrating pharmacist-led medication therapy management with an ASP in a resource-limited clinic. The study revealed a 63.69% reduction in antibiotic prescriptions per 1000 patients over several weeks, suggesting that a pharmacist-led ASP is associated with a significant reduction in antibiotic use in a primary care center serving patients residing in rural or underserved communities. However, a limitation of the study is its pre–post design, which may limit the ability to establish causality [19].

Building on findings from our prior studies and acknowledging the resource constraints faced by rural clinics, we hypothesized that a low-tech approach—specifically, a simplified algorithm-driven antibiotic protocol—could effectively improve antibiotic prescribing practices in rural or underserved settings.

Thus, the objective of our pilot study was to evaluate the impact of an algorithm-driven protocol on antibiotic prescribing in a clinic serving rural or underserved communities.

## 2. Methods

### 2.1. Study Design

This was a pre–post, quasi-experimental study aimed at evaluating antibiotic prescribing practices in a rural healthcare setting. We developed and implemented an algorithm targeting the most prevalent infectious diseases observed in our Federally Qualified Health Center (FQHC): upper respiratory infections (URIs), urinary tract infections (UTIs), and sexually transmitted infections (STIs). Due to the significant challenges and ethical concerns associated with conducting randomized clinical trials in rural or underserved communities—particularly the ethical dilemmas of withholding potentially beneficial intervention from a control group in resource-limited settings—a pre–post design was chosen for this study to ensure that all eligible participants had access to the intervention. This study was approved by the Florida A&M University Institutional Review Board (IRB).

Data collection for the pre-intervention period was conducted from November 2023 to January 2024, while the post-intervention period occurred from March 2024 to May 2024, coinciding with patient visits. To ensure that individual patients were not re-enrolled during multiple visits, the medical record numbers of all enrolled patients were documented for both the pre- and post-intervention periods. This approach maintained the integrity of the dataset by preventing duplicate entries.

### 2.2. Setting and Prior Intervention

The study was conducted at a major local healthcare clinic designated as a Federally Qualified Health Center (FQHC). These federally funded, nonprofit health centers serve medically underserved populations, providing care on a sliding fee scale based on financial need. In 2023, the clinic served 53,824 patients, accounting for approximately 120,818 medical visits. Of these, 56% were covered by Medicaid, 20% were uninsured, 16% had private insurance, and 7% were enrolled in Medicare.

The clinic collaborates with four community pharmacies, all of which participate in the 340B program, ensuring access to affordable medications. Additionally, these pharmacies offer pharmacist-led home health services accredited by the Accreditation Association for Ambulatory Health Care (AAAHC) and provide telehealth services. At the time of the study, the clinic lacked an established antibiotic stewardship program (ASP).

### 2.3. Intervention

The intervention consisted of educational sessions for all healthcare providers, focusing on antibiotic stewardship and the implications of antibiotic resistance, using guidelines and materials provided by the Centers for Disease Control and Prevention (CDC). This educational session was conducted by a pharmacy resident under the supervision of a clinical pharmacist. A simplified antibiotic prescribing algorithm was developed based on Infectious Diseases Society of America (IDSA) and CDC-recommended strategies for the management of URIs, UTIs, and STIs (see Supplementary Materials for the simplified algorithm). This algorithm emphasized appropriate dosing, frequency, and duration and was distributed to all providers to encourage adherence to evidence-based prescribing practices.

### 2.4. Study Population

The study included adult patients at least 18 years old who presented with symptoms consistent with infectious diseases, such as fever, cough, or dyspnea, and who were diagnosed by primary care providers with upper respiratory infections (URIs), urinary tract infections (UTIs), or sexually transmitted infections (STIs). Pediatric patients under the age of 18 were excluded from the analysis, although care was provided to these patients during the study period.

### 2.5. Enrollment

Patients who met the inclusion and exclusion criteria were identified during their primary care visits. During these clinic visits, a pharmacy resident informed eligible participants about the study at the time of enrollment and obtained verbal consent. However, patients were not informed whether they belonged to the pre-intervention or post-intervention group. Data were collected for both the pre- and post-intervention periods.

### 2.6. Outcomes

The primary outcome of the study was to evaluate the change in the proportion of patients receiving guideline-concordant antibiotic prescriptions before and after the intervention. For this study, “guideline-concordant treatment” was defined as adherence to IDSA or CDC-recommended dosing strategies, including appropriate therapy duration, dosing frequency, and strength. Non-concordance was characterized by any deviations in these parameters. Diagnoses were further categorized into UTI, URI, and STI groups for analysis.

### 2.7. Data Collection

To assess the impact of the intervention, patient demographics, diagnoses, and prescription details were recorded in an Excel database. For each prescription, we documented the dose (strength), frequency, and duration. To further evaluate prescribing accuracy, prescription concordance was assessed in relation to patients’ renal function and documented allergies. Data were collected during the pre- and post-intervention periods to evaluate changes in antibiotic prescribing patterns and identify potentially inappropriate prescriptions. Of note, in this study, neither the researchers nor the pharmacy resident assessed the accuracy of the diagnoses. Instead, all prescription evaluations were based solely on the diagnoses documented in the electronic health record by the prescribing clinician.

### 2.8. Statistical Analysis

To assess the impact of the intervention, antibiotic prescriptions for eligible patients during the post-intervention period were compared to those for eligible patients during the pre-intervention period. Descriptive statistics were reported as counts and percentages to summarize patient demographics at the time of their primary care visits. To assess whether the intervention was associated with a significant change in guideline-concordant antibiotic prescribing, a Chi-square test for independence was conducted. This test compared the proportions of guideline-concordant prescriptions between the pre- and post-intervention periods, as these groups were independent. The odds ratio and 95% confidence interval for potentially inappropriate antibiotic prescriptions will be reported comparing pre- and post-intervention periods. A priori, statistical significance was defined as *p* < 0.05 using a two-tailed test. This analysis was conducted with IBM SPSS Statistics version 29.0.2.0 (20).

## 3. Results

A total of 201 patients participated in this pilot study, with 101 patients in the pre-intervention group and 100 patients in the post-intervention group. Among those in the pre-intervention group, 77% of the participants were female, compared to 72% in the post-intervention group. Regarding racial demographics, 47% of the pre-intervention group identified as African American, and 41% identified as White. In the post-intervention group, 46% were African American, and 33% were White. Additional demographic details are presented in Table 1.

Following the intervention, the proportion of patients receiving potentially inaccurate antibiotic prescriptions—defined as deviations from guideline-concordant recommendations—decreased by 12.6%, from 37.6% in the pre-intervention group to 25.0% in the post-intervention group. Chi-square analysis indicated a numerical decrease approaching statistical significance (χ^2^ = 3.72, *p* = 0.054). The odds ratio (OR) from pre- to post-intervention periods was 0.55 (95% CI: 0.30–1.01). Table 2 presents all types of errors categorized by the type of infectious disease.

## 4. Discussion

In this pilot study, we observed the potential benefits of implementing an algorithm-driven protocol to improve antibiotic prescribing in clinics with limited resources to fully adopt antimicrobial stewardship programs (ASP). Following the protocol’s introduction, there was an improvement in guideline-concordant prescribing practices. Although the reduction in inappropriate prescriptions (12.6%) did not reach statistical significance during the study’s short duration, the numerical decrease suggests that such interventions can positively influence prescribing behavior. In a similar study conducted in an urgent care setting, Lee et al. (2022) observed significant improvements after implementing outpatient antimicrobial stewardship guidelines [20]. Their intervention, which included provider education and pocket guides, increased guideline-concordant prescribing from 50% to 70% (*p* < 0.001) and reduced the duration of antibiotic prescriptions for UTIs from 7 days to 5 days (*p* = 0.007). These findings align with our hypothesis, suggesting that targeted interventions, even with minimal resources, can enhance antibiotic stewardship. Extending the duration of our study could provide a clearer understanding of the long-term benefits, but the observed results underscore the potential role of algorithm-driven protocols in improving prescribing practices in resource-limited settings.

While prescribing practices for urinary tract infections (UTIs) improved, discrepancies persisted in guideline adherence for upper respiratory infections (URIs) and sexually transmitted infections (STIs). These results suggest that while the intervention was beneficial for certain conditions, targeted, condition-specific strategies may be necessary to align prescribing practices with recommended guidelines across all infectious disease categories. The small sample size and short study duration likely limited our ability to fully observe the intervention’s impact.

### 4.1. Implications for Public Health

Our study is significant as it contributes to the growing body of literature on antimicrobial stewardship in rural primary care settings. It also emphasizes the vital role primary care practices play in influencing the rate of AMR through their antibiotic prescribing practices. Yau et al. (2021), in their narrative review, collated evidence of the correlations between prescribing patterns in rural primary care centers and increased AMR. For instance, azithromycin use was associated with nasal carriage of *S. pneumoniae* and *S. aureus* strains resistant to macrolides [21,22,23]. Similarly, Hare et al. identified a dose–response relationship between azithromycin use and the carriage of macrolide-resistant *S. pneumoniae* and *S. aureus* [22]. Furthermore, Costelloe et al. emphasized this connection, demonstrating that multiple or prolonged antibiotic courses, particularly with amoxicillin and trimethoprim, are linked to higher AMR rates [21,24]. These findings underscore the urgent need for optimized antibiotic stewardship in rural primary care settings to reduce AMR and preserve the efficacy of treatments.

Implementing antibiotic stewardship programs (ASPs) in rural or underserved communities offers a valuable opportunity to reduce the spread of antibiotic-resistant organisms. Our experience provides a practical model for ASP implementation in resource-limited settings, demonstrating that even small-scale interventions can drive meaningful changes. With sustained efforts, we anticipate that this tailored approach will prove both sustainable and impactful in improving antimicrobial use in such settings.

### 4.2. Strengths and Limitations

The strength of our study lies in the use of evidence-based, algorithm-driven strategies tailored to the unique needs of a resource-limited clinic. However, our findings must be interpreted in light of the study’s limitations, including the small sample size and short duration, which may have contributed to the lack of statistically significant differences observed, potentially leading to a type II error. These factors limit the generalizability of our results. Additionally, the inherent limitations of the pre–post study design, such as its inability to account for unmeasured confounding variables or external events that may have influenced outcomes during the study period, further impact the robustness of our findings. However, given the constraints of our setting, the pre–post design was the most practical choice to ensure inclusivity and avoid excluding individuals. Despite these limitations, our study provides meaningful insights for those aiming to implement and adapt ASPs in rural or resource-constrained environments, contributing to the broader understanding of effective strategies to address antimicrobial resistance in these settings.

### 4.3. Future Directions

In this study, we did not assess the appropriateness of antibiotic selection but focused solely on evaluating the concordance of prescribed antibiotics with clinical guidelines based on the documented diagnosis. Future studies will aim to evaluate the effect of the algorithm on the appropriateness of antibiotic selection. As we continue this study, we will further refine our intervention to improve antibiotic prescribing practices and document the number of providers who used the simplified algorithm in practice through survey. We anticipate that as we extend the study’s duration and increase the sample size, we will be able to more effectively evaluate the long-term outcomes and the sustainability of the intervention. Additionally, as this current study does not have a control group, future studies should consider conducting cluster analyses involving multiple rural clinics, with some clinics implementing an educational intervention (with the algorithm) while others serve as controls. Our overarching goal is to enhance the implementation and effectiveness of ASPs in resource-limited clinics.

## 5. Conclusions

This pilot study demonstrates the potential of algorithm-driven antibiotic stewardship protocols in rural and underserved settings. While the observed improvement in guideline-concordant prescribing practices was promising, further research with larger sample sizes and longer study periods is necessary to confirm these findings. Our experience underscores the importance of implementing ASPs tailored to the unique needs of resource-limited settings, with the potential to significantly impact antimicrobial resistance on a broader scale.

## Figures and Tables

**Table 1:** Adult Study Participants Characteristics.

Demographics	Pre-Group (N = 101)	Post-Group (N = 100)
**Gender**		
Female	77 (76.2%)	72 (71.3%)
Male	24 (23.8%)	28 (27.7%)
**Race/Ethnicity**		
African American	47 (46.5%)	46 (45.5%)
White	41 (40.5%)	33 (32.7%)
Hawaiian	1 (1.0%)	2 (2.0%)
Hispanic	1 (1.0%)	3 (3.0%)
Alaskan	0 (0.0%)	1 (1.0%)
American Indian	0 (0.0%)	1 (1.0%)
Unknown	11 (11%)	14 (13.9%)
**Age**		
18–24	18 (17.8%)	23 (23.0%)
25–34	33 (32.7%)	27 (27.0%)
35–44	21 (20.8%)	16 (15.8%)
45–54	13 (12.9%)	18 (18.0%)
55–64	12 (11.9%)	12 (12.0%)
65 and older	4 (4.0%)	3 (3.0%)
Unknown	0 (0.0%)	1 (1.0%)
**Insurance**		
Private	33 (32.7%)	33 (32.7%)
Medicare	7 (7.0%)	7 (7.0%)
Medicaid	27 (27.0%)	27 (27.0%)
Uninsured	34 (33.7%)	34 (34.0%)
**Diagnosis**		
Chlamydia	12 (11.9%)	8 (8.0%)
Chlamydia/Trichomonas	1 (1.0%)	0 (0.0%)
Gonorrhea	7 (7.0%)	5 (5.0%)
Gonorrhea/Trichomonas	2 (2.0%)	1 (1.0%)
Trichomonas	17 (16.8%)	13 (13.0%)
Syphilis	3 (3.0%)	2 (2.0%)
Urinary Tract Infection	20 (19.8%)	16 (16.0%)
Pharyngitis	23 (22.8%)	24 (24.0%)
Sinusitis	16 (15.8%)	20 (20.0%)
Gonorrhea/Chlamydia	0 (0.0%)	2 (2.0%)
Gonorrhea/Chlamydia/Syphilis	0 (0.0%)	1 (1.0%)
Urinary Tract Infection/Trichomonas	0 (0.0%)	1 (1.0%)
Pharyngitis/Trichomonas	0 (0.0%)	1 (1.0%)
CAP	0 (0.0%)	2 (2.0%)
Upper Respiratory	0 (0.0%)	4 (4.0%)

**Table 2:** The number of patients with at least one prescription that is discordant with clinical guidelines—before and after intervention.

Type of Discrepancy	Pre-Intervention (N = 101)	Post-Intervention (N = 100)
**All [Irrespective of diagnosis]**	38 *	25 *
All—Duration	23	10
All—Wrong ABX	17	12
All—Strength	2	1
All—Frequency	2	1
All—ABX not recommended	9	6
**UTI (All Patients)**	19/20	4/17
UTI Duration	15/20	3/17
UTI Wrong ABX	4/20	2/17
UTI Strength	1/20	0/17
UTI Frequency	2/20	1/17
UTI ABX not recommended	0/20	0/17
**URI (All Patients)**	16/39	13/49
URI Duration	6/39	4/49
URI Wrong ABX	4/39	2/49
URI Strength	0/39	1/49
URI Frequency	0/39	0/49
URI ABX not recommended	7/39	6/49
**STI (All Patients)**	4/35	7/42
STI Duration	3/35	3/42
STI Wrong ABX	1/35	4/42
STI Strength	1/35	0/42
STI Frequency	0/35	0/42
STI ABX not recommended	1/35	0/42

Note: A single patient may have more than one type of prescription discrepancy. ABX: antibiotics. * (χ^2^ = 3.72, OR 0.55 [95% CI: 0.30–1.01], *p* = 0.054).

## Data Availability

Data will be made available upon reasonable request following the institutional applicable guidelines and approvals.

## Supplementary Materials

The following supporting information can be downloaded at https://www.mdpi.com/article/10.3390/pharmacy13010030/s1: simplified antibiotic prescribing algorithm.
