# Supplementary material for: A Pilot Study Evaluating the Impact of an Algorithm-Driven Protocol on Guideline-Concordant Antibiotic Prescribing in a Rural Primary Care Setting
Source: Pharmacy (Basel). 2025 Feb 19;13(1):30. doi: 10.3390/pharmacy13010030 (PMC11859786; doi:10.3390/pharmacy13010030)
Supplement: Supplementary file 1 [file pharmacy-13-00030-s001.zip › pharmacy-3429175-supplementary.pdf]

Locally- Adapted Simplified Treatment Algorithm for Acute Respiratory Infections, and Sexually Transmitted Infections, Urinary Tract Infections

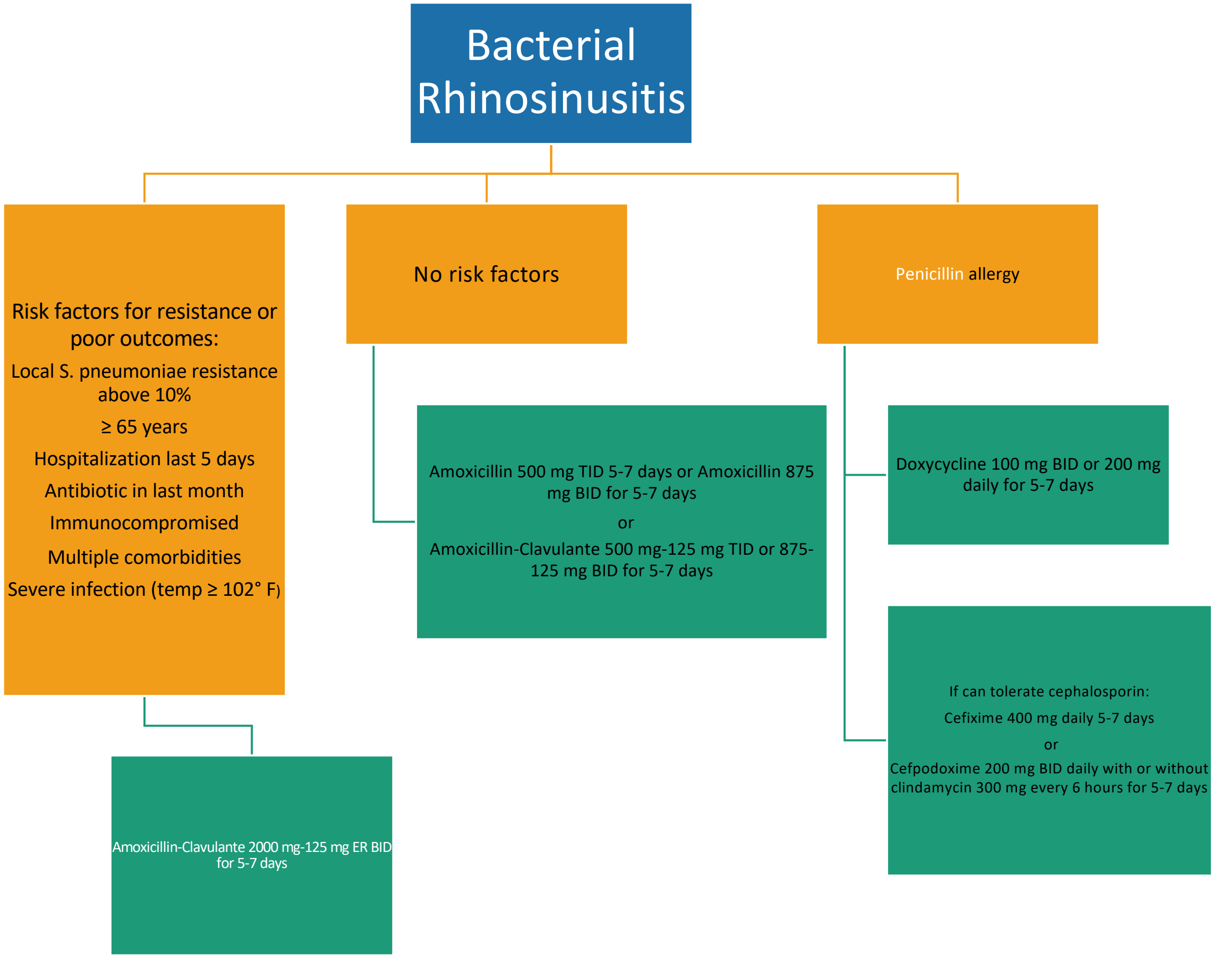
Bacterial Rhinosinusitis
Risk factors for resistance or poor outcomes:
Local S. pneumoniae resistance above 10%
≥ 65 years
Hospitalization last 5 days
Antibiotic in last month
Immunocompromised
Multiple comorbidities
Severe infection (temp ≥ 102° F)
Amoxicillin-Clavulante 2000 mg-125 mg ER BID for 5-7 days
No risk factors
Amoxicillin 500 mg TID 5-7 days or Amoxicillin 875 mg BID for 5-7 days
or
Amoxicillin-Clavulante 500 mg-125 mg TID or 875-125 mg BID for 5-7 days
Penicillin allergy
Doxycycline 100 mg BID or 200 mg daily for 5-7 days
If can tolerate cephalosporin:
Cefixime 400 mg daily 5-7 days
or
Cefpodoxime 200 mg BID daily with or without clindamycin 300 mg every 6 hours for 5-7 days

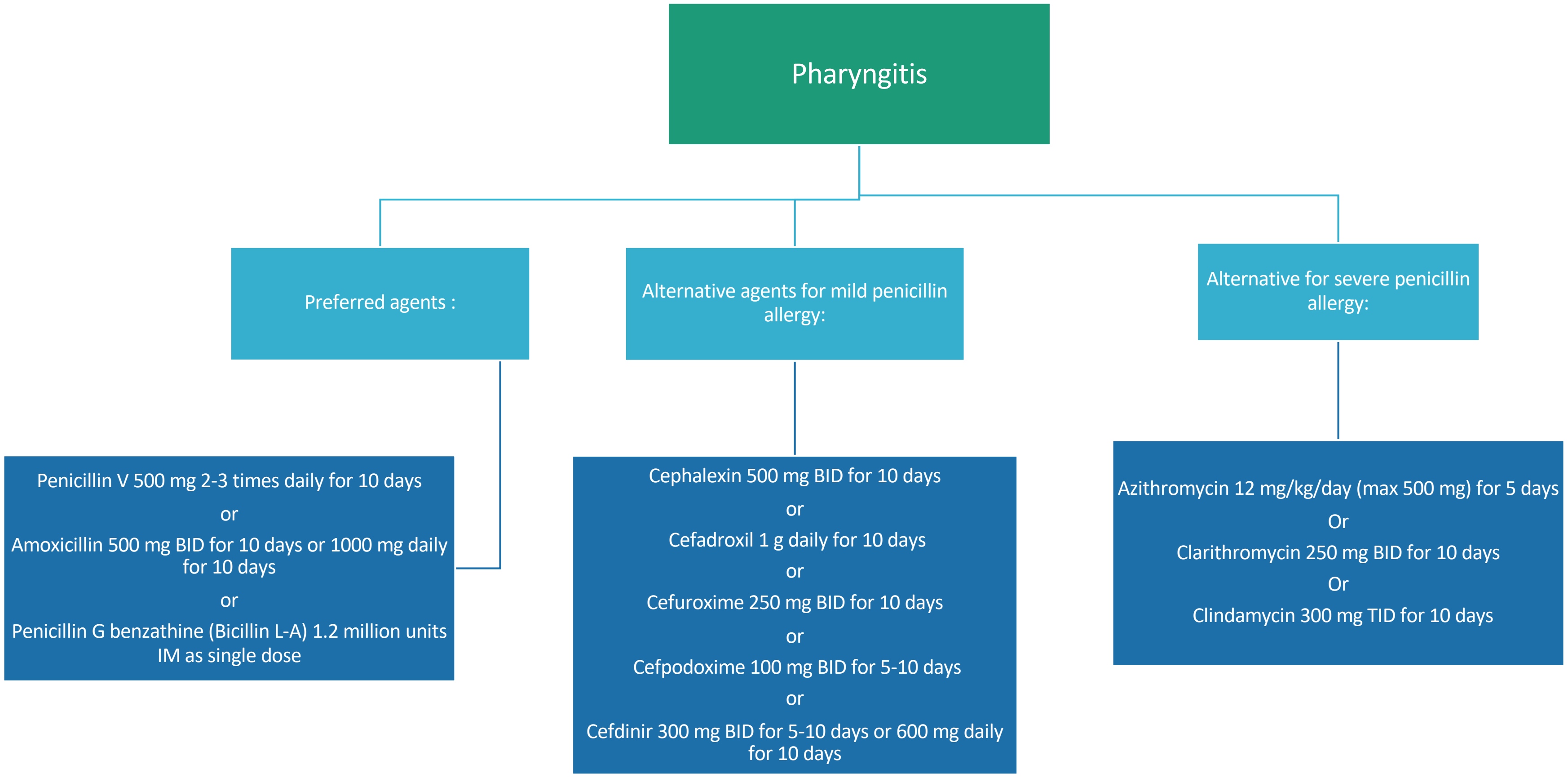
Pharyngitis
Preferred agents :
Alternative agents for mild penicillin allergy:
Alternative for severe penicillin allergy:
Penicillin V 500 mg 2-3 times daily for 10 days
or
Amoxicillin 500 mg BID for 10 days or 1000 mg daily for 10 days
or
Penicillin G benzathine (Bicillin L-A) 1.2 million units IM as single dose
Cephalexin 500 mg BID for 10 days
or
Cefadroxil 1 g daily for 10 days
or
Cefuroxime 250 mg BID for 10 days
or
Cefpodoxime 100 mg BID for 5-10 days
or
Cefdinir 300 mg BID for 5-10 days or 600 mg daily for 10 days
Azithromycin 12 mg/kg/day (max 500 mg) for 5 days
Or
Clarithromycin 250 mg BID for 10 days
Or
Clindamycin 300 mg TID for 10 days

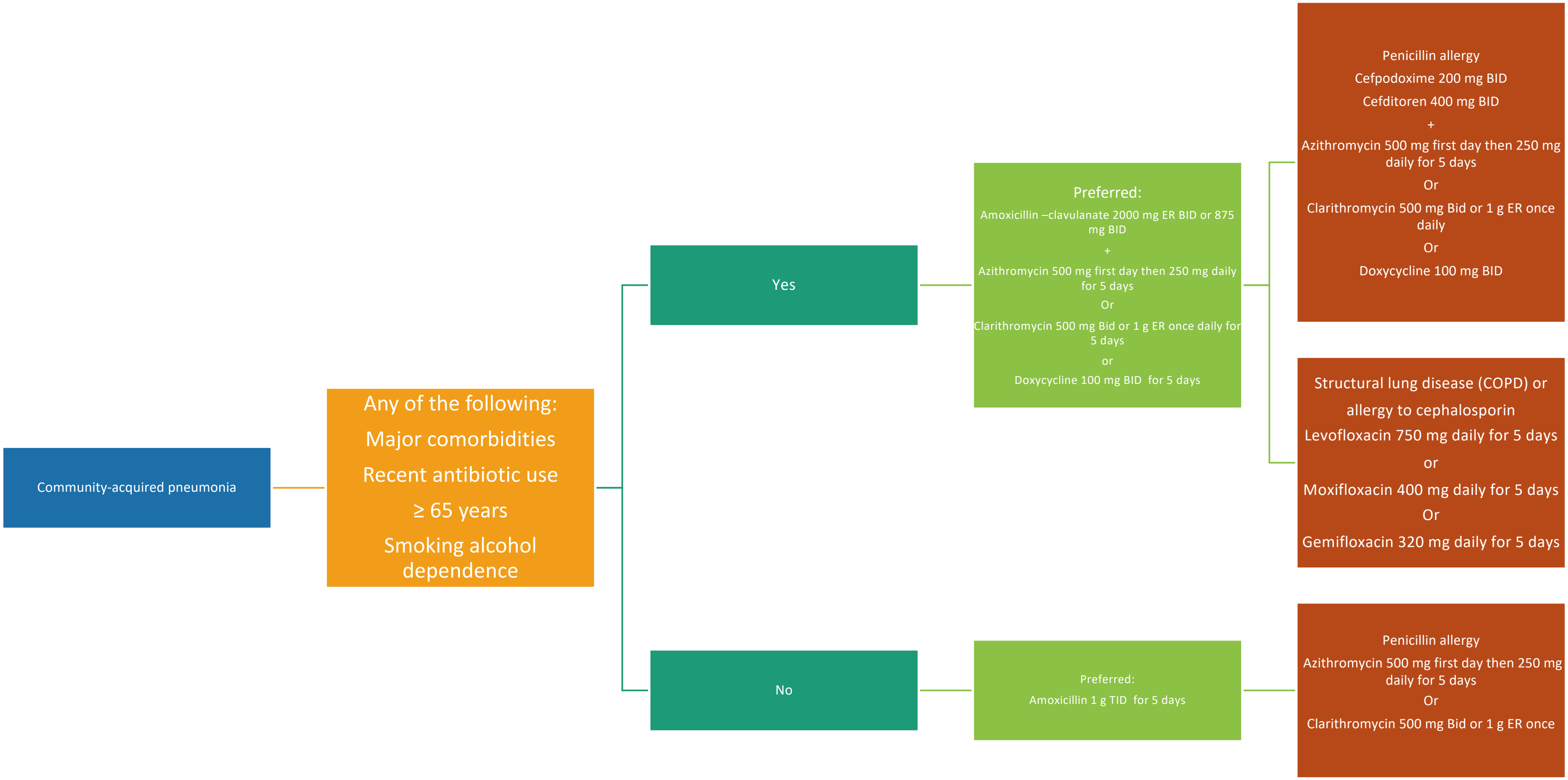
Community-acquired pneumonia
Any of the following:
Major comorbidities
Recent antibiotic use
≥ 65 years
Smoking alcohol dependence
Yes
No
Preferred:
Amoxicillin –clavulanate 2000 mg ER BID or 875 mg BID
+
Azithromycin 500 mg first day then 250 mg daily for 5 days
Or
Clarithromycin 500 mg Bid or 1 g ER once daily for 5 days
or
Doxycycline 100 mg BID for 5 days
Penicillin allergy
Cefpodoxime 200 mg BID
Cefditoren 400 mg BID
+
Azithromycin 500 mg first day then 250 mg daily for 5 days
Or
Clarithromycin 500 mg Bid or 1 g ER once daily
Or
Doxycycline 100 mg BID
Structural lung disease (COPD) or allergy to cephalosporin
Levofloxacin 750 mg daily for 5 days
or
Moxifloxacin 400 mg daily for 5 days
Or
Gemifloxacin 320 mg daily for 5 days
Preferred:
Amoxicillin 1 g TID for 5 days
Penicillin allergy
Azithromycin 500 mg first day then 250 mg daily for 5 days
Or
Clarithromycin 500 mg Bid or 1 g ER once

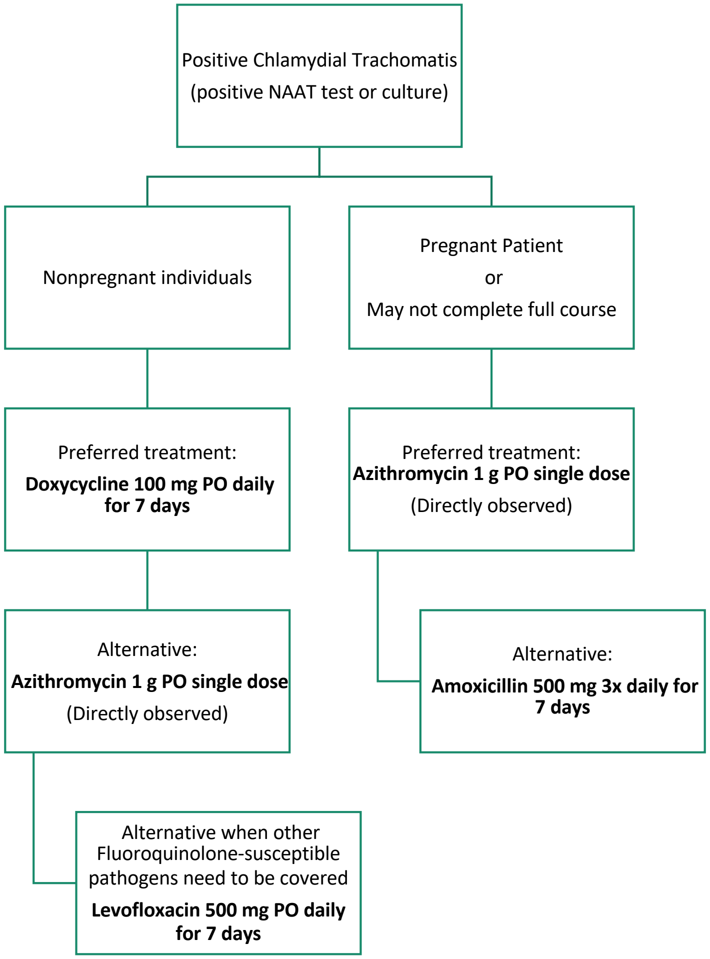
Positive Chlamydial Trachomatis
(positive NAAT test or culture)
Nonpregnant individuals
Pregnant Patient
or
May not complete full course
Preferred treatment:
Doxycycline 100 mg PO daily for 7 days
Preferred treatment:
Azithromycin 1 g PO single dose
(Directly observed)
Alternative:
Azithromycin 1 g PO single dose
(Directly observed)
Alternative:
Amoxicillin 500 mg 3x daily for 7 days
Alternative when other Fluoroquinolone-susceptible pathogens need to be covered
Levofloxacin 500 mg PO daily for 7 days

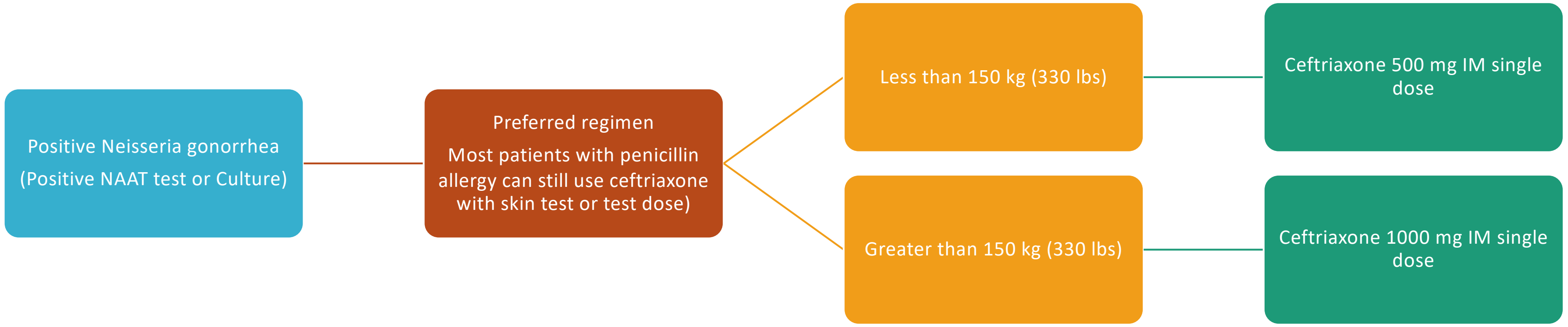
Positive Neisseria gonorrhea
(Positive NAAT test or Culture)
Preferred regimen
Most patients with penicillin allergy can still use ceftriaxone with skin test or test dose)
Less than 150 kg (330 lbs)
Greater than 150 kg (330 lbs)
Ceftriaxone 500 mg IM single dose
Ceftriaxone 1000 mg IM single dose

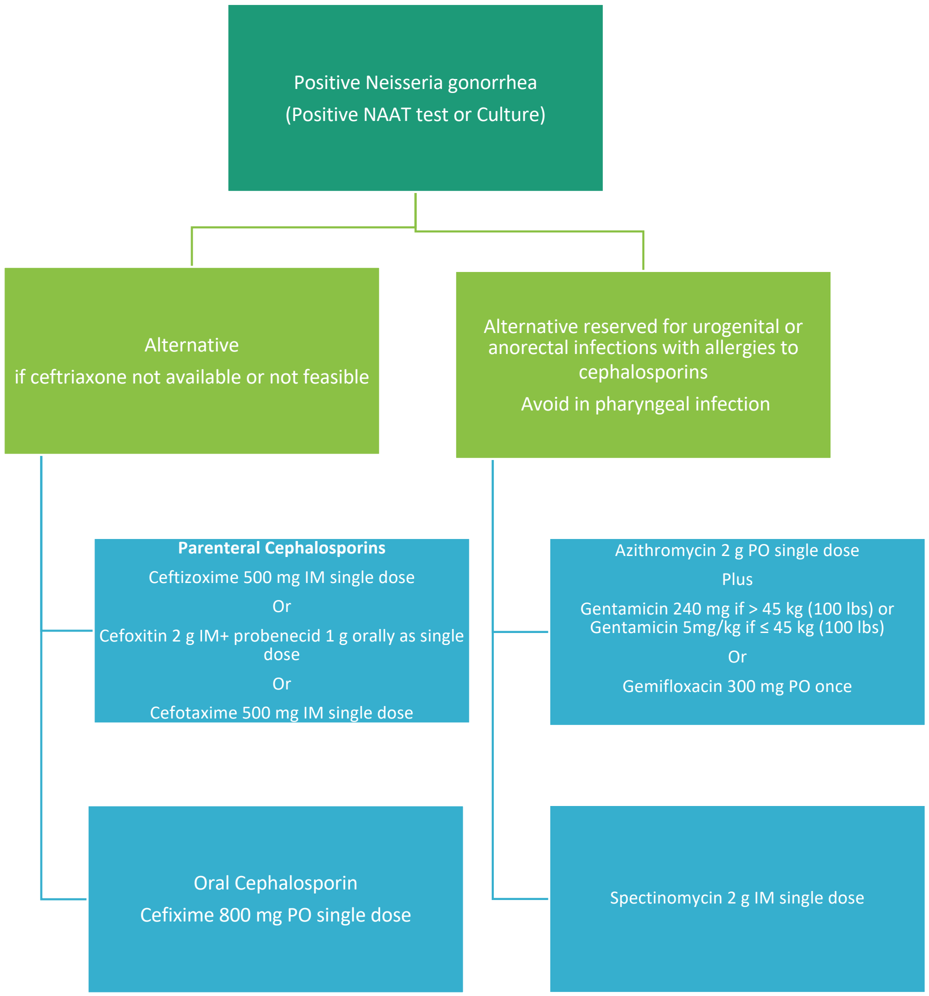
Positive Neisseria gonorrhea
(Positive NAAT test or Culture)
Alternative
if ceftriaxone not available or not feasible
Alternative reserved for urogenital or anorectal infections with allergies to cephalosporins
Avoid in pharyngeal infection
Parenteral Cephalosporins
Ceftizoxime 500 mg IM single dose
Or
Cefoxitin 2 g IM+ probenecid 1 g orally as single dose
Or
Cefotaxime 500 mg IM single dose
Oral Cephalosporin
Cefixime 800 mg PO single dose
Azithromycin 2 g PO single dose
Plus
Gentamicin 240 mg if > 45 kg (100 lbs) or Gentamicin 5mg/kg if ≤ 45 kg (100 lbs)
Or
Gemifloxacin 300 mg PO once
Spectinomycin 2 g IM single dose

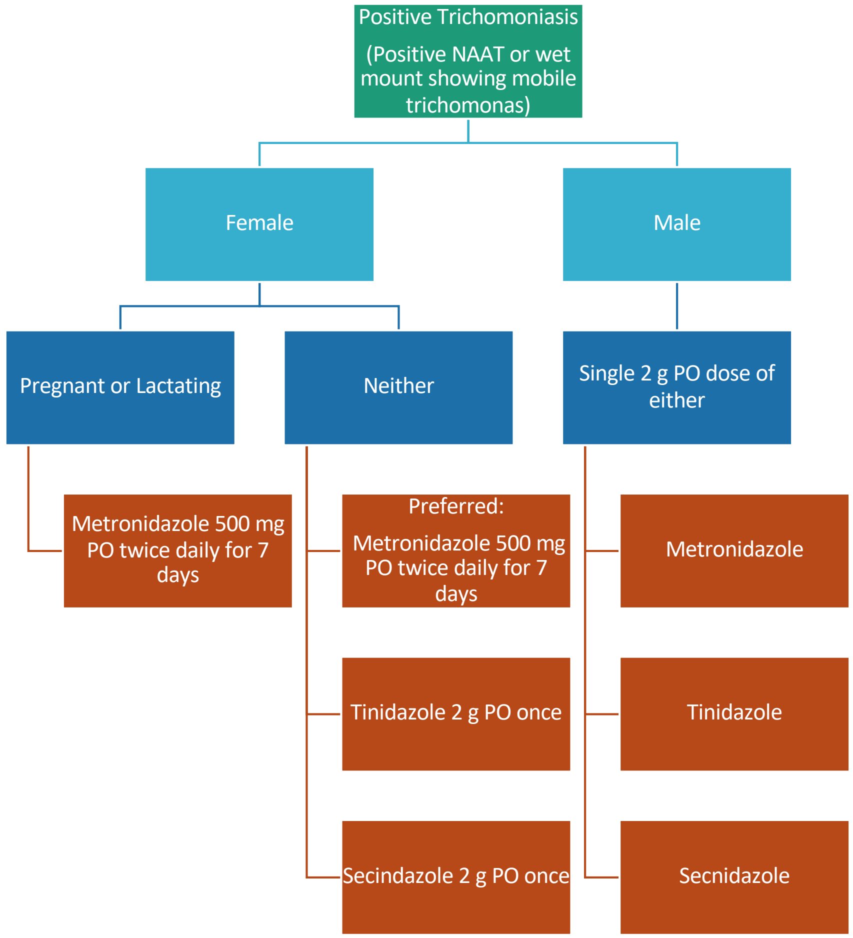
Positive Trichomoniasis
(Positive NAAT or wet mount showing mobile trichomonas)
Female
Male
Pregnant or Lactating
Neither
Single 2 g PO dose of either
Metronidazole 500 mg PO twice daily for 7 days
Preferred:
Metronidazole 500 mg PO twice daily for 7 days
Tinidazole 2 g PO once
Secindazole 2 g PO once
Metronidazole
Tinidazole
Secnidazole

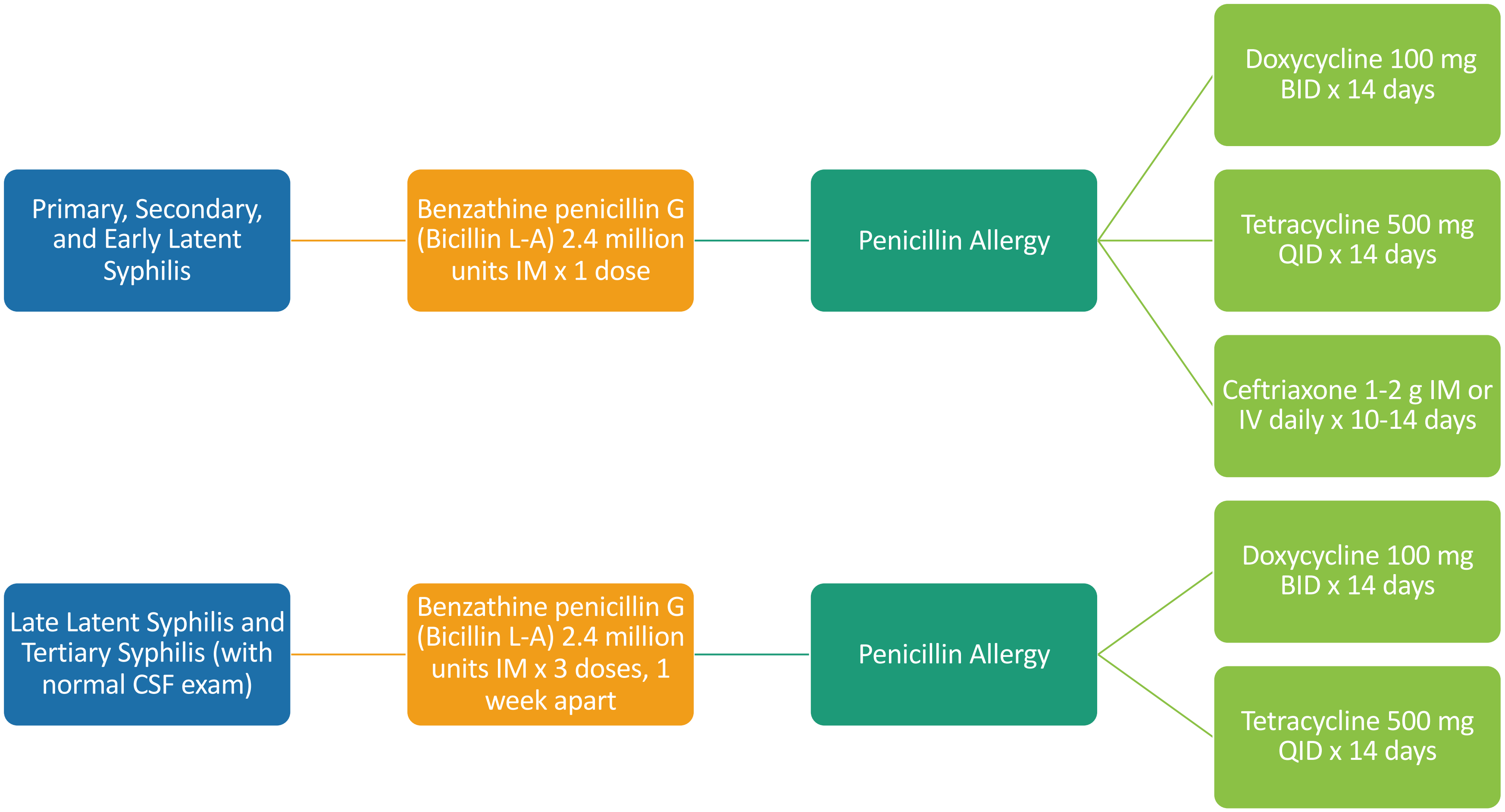
Primary, Secondary, and Early Latent Syphilis
Benzathine penicillin G (Bicillin L-A) 2.4 million units IM x 1 dose
Penicillin Allergy
Doxycycline 100 mg BID x 14 days
Tetracycline 500 mg QID x 14 days
Ceftriaxone 1-2 g IM or IV daily x 10-14 days
Late Latent Syphilis and Tertiary Syphilis (with normal CSF exam)
Benzathine penicillin G (Bicillin L-A) 2.4 million units IM x 3 doses, 1 week apart
Penicillin Allergy
Doxycycline 100 mg BID x 14 days
Tetracycline 500 mg QID x 14 days

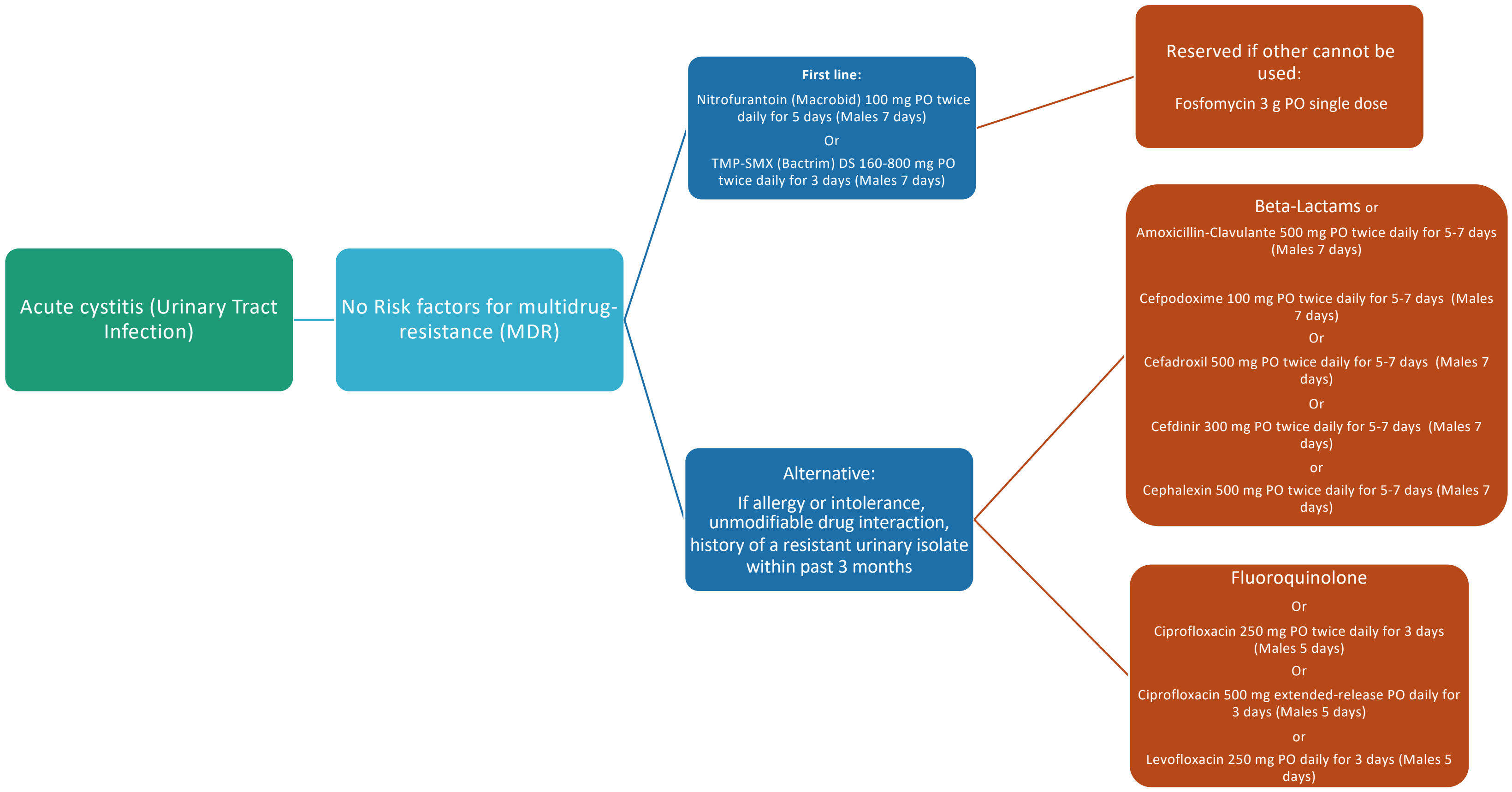
Acute cystitis (Urinary Tract Infection)
No Risk factors for multidrug-resistance (MDR)
First line:
Nitrofurantoin (Macrobid) 100 mg PO twice daily for 5 days (Males 7 days)
Or
TMP-SMX (Bactrim) DS 160-800 mg PO twice daily for 3 days (Males 7 days)
Reserved if other cannot be used:
Fosfomycin 3 g PO single dose
Alternative:
If allergy or intolerance, unmodifiable drug interaction, history of a resistant urinary isolate within past 3 months
Beta-Lactams or
Amoxicillin-Clavulante 500 mg PO twice daily for 5-7 days (Males 7 days)
Cefpodoxime 100 mg PO twice daily for 5-7 days (Males 7 days)
Or
Cefadroxil 500 mg PO twice daily for 5-7 days (Males 7 days)
Or
Cefdinir 300 mg PO twice daily for 5-7 days (Males 7 days)
or
Cephalexin 500 mg PO twice daily for 5-7 days (Males 7 days)
Fluoroquinolone
Or
Ciprofloxacin 250 mg PO twice daily for 3 days (Males 5 days)
Or
Ciprofloxacin 500 mg extended-release PO daily for 3 days (Males 5 days)
or
Levofloxacin 250 mg PO daily for 3 days (Males 5 days)
